# My Experiences in a Field Ambulance

**Published:** 1914-10-24

**Authors:** 


### MY EXPERIENCES IN A FIELD AMBULANCE.

### By an Officer of the British Expeditionary Force.

The present campaign is the first opportunity that has occurred of testing in actual practice the working of the existing system of field ambulances. In other words, these medical units are in the nature of an experiment, now on trial in actual warfare for the first time. Naturally enough, experience has shown that there are many details connected with their interior organisation in which alterations are advisable; and doubtless many improvements will be brought about when the lessons of the campaign have been fully considered and digested. Nor is it alone in matters of detail that modifications of the present scheme will be necessary; even new principles will have to be adopted in some important respects, though it is, of course, true that there will be less to reconstruct here than in numberless minutiæ.

### "Some Magnificent Work."

It would be idle to attempt at the present moment to indicate the nature of the changes in field-ambulance organisation which seem desirable. For one thing, it would be premature to discuss the matter now; for another, any expression of opinion would be solely that of an individual, and might not command universal assent from the officers of the R.A.M.C.; and, finally, it might not be judicious at this stage of the war to criticise arrangements which cannot be altered now, but have to be made the best of. Such criticism, too, might be misunderstood at home; so let it be plainly stated at once that the field ambulances have already done magnificent work, and that this is not merely the biassed opinion of one who works in one of them, but reflects the views of every combatant officer and man who has had opportunities of judging.

Instead, therefore, of devoting my description to the working of a field ambulance as developed in the war, let me rather retail a few disconnected incidents and reminiscences, professional and other, which occur to the memory. In order to comply with censorship requirements, no place names will be given, but there can be no harm in stating that they range from Belgium down to the latitude of Paris and back again as far as the north side of the Aisne.

### The Organisation of a Division.

A Division is a self-contained force of rather over 18,000 men (in the British Army), complete within itself in every way. It contains three Brigades (twelve battalions) of infantry, its own artillery, engineers, supply columns, ammunition trains, and so forth; it also includes three field ambulances, the composition and interior organisation of which have frequently been described on past occasions in The Hospital. It will be noted that there is a field ambulance for each Brigade, and the former are numbered correspondingly with the latter; thus the First Division includes the 1st, 2nd, and 3rd Brigades, and also the 1st, 2nd, and 3rd Field Ambulances; the Second Division has the 4th, 5th, and 6th Brigades, and also the 4th, 5th, and 6th Field Ambulances; and similarly for the others. As a general rule, each field ambulance works with the correspondingly numbered Brigade, collects its wounded, and bivouacs in the same camp. But this arrangement is only for convenience, and is not hard and fast. The General of the Division can send all three ambulances to one Brigade if he thinks fit, or he can detach them from the corresponding Brigades altogether and encamp them elsewhere. Further, each ambulance consists of three sections, each complete and capable of being sent on detached duty. In any case, every ambulance collects and treats, of course, any wounded that are encountered, irrespective of the Division or Brigade to which they belong; irrespective, indeed, of whether they are English or German. These technical names explained, let me proceed at last with my tale.

### Night Marches During the Retreat.

During the great retreat from Belgium it was of prime importance that the main roads should be kept clear for the use of the fighting rearguard, so that when the latter could hold its ground no longer it could retreat rapidly to take up a new position. In order to keep these roads clear, side roads and lanes of all sorts were allotted to troops not actually part of the striking force. It thus happened that R.A.M.C. units (and other troops not actually fighting) were frequently sent long détours in semicircles or sinuous curves, and thus were compelled to march much longer distances than the infantry, artillery, and cavalry. This was, of course, perfectly correct, and not one of the R.A.M.C. grumbled at it; for, obviously, the far lighter load carried by R.A.M.C. soldiers was abundant compensation for the longer marches. A great many of these long and weary marches were done at night—frequently all night—and the drivers of waggons often fell asleep on their boxes, as also did men on horseback, or even on foot.

### Dodging Fatigue.

To spare the horses as much as possible all waggon orderlies (who usually expect to be allowed to ride on the box, or else inside the ambulance waggon) were ordered to march, each one behind his own waggon. A good many instances occurred of tired infantry men, who had fallen out from their regiments owing to sore feet, fatigue, exhaustion, or lack of pluck, attempting to "get a lift" on the ambulance waggons. This the waggon orderlies were supposed to prevent, but they did not always succeed in doing so. The favourite dodge was for a man to climb into an ambulance waggon on the excuse that one of the medical officers had ordered him to do so. Asked for the name of the officer, he would reply that he did not know (how should he, especially in pitch darkness?); to verify or disprove this statement would involve riding or walking up and down the whole column and interviewing every officer in turn. Sometimes the statement would be found to be true; more often false. One man who was turned out of a waggon (after having his name and regiment taken) in such circumstances immediately afterwards shot his right forefinger off with his own rifle; this proved that he was determined to march no further, but it was, of course, a serious military offence.

### A Night in an Old Chateau.

Many of the places through which the army passed were quite clearly of great antiquity. A large percentage of the churches in some districts are 600, 700, and 800 years old; some of them (apart altogether from Rheims Cathedral and other well-known fanes) have been more or less shattered by German shell fire, but many have escaped. On one occasion the writer spent the night in a fine old fourteenth-century château, now converted into a farm. Probably the château was already a farm before the Revolution, and thus escaped being burned. At any rate, it was in a fine state of preservation, with a large arched crypt, wide spiral stone staircase, central square tower, and enormous rooms. In the kitchen was a huge iron back to the fireplace, with a coat of arms in relief surmounted by a coronet. Another farm which was visited was evidently of ecclesiastical origin; the barn was an old Gothic church, with all the pointed arches filled in with stone, but clearly visible. This was a fifteenth-century erection.

### A Field Ambulance Bombarded.

On one occasion, during a battle, the field ambulance was drawn up in an orchard, behind a wood, which prevented the enemy from seeing it. Suddenly shells began to burst in and beyond the orchard, and for a few minutes there was considerable risk of being hit. However, as luck would have it, no damage was done, and presently the shells began to fall further along the edge of the wood, about two or three hundred yards away. As there were no British troops there, it was difficult to make out why the Germans were wasting so much ammunition; for they kept up a really vigorous bombardment. Later on the problem was solved, for it appeared that a broken country cart behind a haycock was mistaken, quite excusably, by the German artillerymen for a gun, and that they had shelled it for a long time before their error dawned on them. On this same day, and close at hand, they dropped a lot of shells into the adjacent village. Another field ambulance happened to be in the main street at the time, and encountered two of the shells, losing six men killed and nine wounded. This was about two hundred yards from the incident of the orchard, but was not a deliberate case of firing on the Red Cross.

### Running the Gauntlet of Shrapnel Fire.

On another occasion the writer was sent to take a party of bearers as near as possible to a certain bridge which was under very heavy fire, and to pick up any casualties which might occur. By using such cover as existed, it was quite easy to gain the shelter of four large haystacks in a flat meadow alongside the river, about 300 or 400 yards from the bridge. Through the meadow ran a road to the bridge, and along this road our cavalry were running the gauntlet of the German shrapnel. In ones and twos they would appear at one edge of the open stretch, and gallop furiously for shelter to the other side. Each one as he appeared would be made the target for at least one shell, and to the R.A.M.C., lying snug under the haystacks, the sight was most absorbing. The German gunners had very little luck that day: one shell would burst thirty yards too far; the next fifty yards too short; the next, on the road, but just behind the gallopers; the next, just in front of the man aimed at. In three hours they hit only one man: his horse was killed, but he was wounded in the foot alone, and before the bearer party could get to him he had managed to reach safety. When the shrapnel was very short, as it sometimes was, it burst right alongside the haystacks; but as this kind of shell carries forwards, and not at all backwards, one had only to stay under the lee of the stacks to be fairly safe.

### A Plucky French Woman.

Another exciting—almost too exciting—experience was in a small town, one end of which was being knocked to pieces by high-explosive shells. The other end of the town was fairly safe, and three or four houses were being used as a dressing station for the wounded. Word was brought in by a Frenchwoman that a wounded British soldier was lying in a house at the far end of the town, so there was nothing for it but to go and see him. Concealing one's natural trepidation as much as possible, and guided by the plucky Frenchwoman, who seemed quite unconcerned by the ruins all about, I took a satchel of dressings and went to find the wounded man. On the way a terrific report and a dense cloud of black dust and smoke just ahead showed where a percussion shell of high explosive had landed right in the middle of the road, not more than twenty-five yards away. On arriving at the house, one of three or four left standing in a street of a dozen or so, it was found that the injured man was not a soldier at all, but a French civilian! Fortunately he was not badly hurt, so after dressing his (two) wounds a retreat was beaten to a safer neighbourhood. The reason for the erroneous information was never discovered, but presumably the plucky (though mendacious) Frenchwoman thought that her countryman was more likely to be succoured if she represented him to be a British soldier. It was in this same town that several children were injured by bits of shell or by splinters of masonry; it seemed impossible to keep them indoors and out of harm's way in the deep cellars where most of the inhabitants took refuge.

On one occasion I had been to see two of these wounded children and received four eggs as a fee, and a most welcome fee too. I had at this time charge of over a hundred wounded in the parish church, and I offered the eggs to several of the officers and men. Not a single one would accept one, each saying there must be others in the church more in need of the luxury than he was.

### OUR HEROIC WOUNDED.

And, indeed, I cannot close these few and fragmentary impressions without a tribute to the British soldier when wounded. On the field and in his trenches he has, as the world knows by this time, displayed a dogged valour, a stubborn endurance, and an aptitude for fighting which are worthy of his ancestors and of the best traditions of the Army. Glorious as are the laurels he has won there, they are at least equalled by those due to his magnificent heroism in the hospitals, in the ambulances, and when lying wounded on the field. It is the rarest possible thing to hear as much as a groan from the wounded, even from those with the fearful compound and comminuted fractures, of which there have been a good many. The splendid stoicism of officers and men when wounded has deeply impressed the French, who are inclined (despite their fine military qualities in many other respects) to make a fearful fuss over the most trivial injuries. I have repeatedly heard from the French tributes of the most profound admiration for this trait of the British soldier, notably from the fine old *curé* of the church before mentioned, in which a large number of our wounded were being treated. This excellent priest was unwearied in good works for our men, and rendered most valuable assistance in every way: he even himself removed the big candles from the high altar in order to use them for lighting the body of the church where the wounded were lying. His beautiful thirteenth-century church was unfortunately later on bombarded by the Germans, who made four or five big holes in the walls with their high-explosive shells. He was as calm under fire as the most experienced soldier, which renders his volunteered and repeated testimony to the British soldier all the more valuable and impressive.

